# Clinical outcomes of NBF gel application in managing mucositis associated with xerostomia

**DOI:** 10.1186/s40902-024-00445-6

**Published:** 2024-09-29

**Authors:** György Szabó, Zsolt Németh, Márton Kivovics

**Affiliations:** 1 Department of Oro-Maxillofacial Surgery and Stomatology, Semmelweis University, Mária Utca 52, Budapest, 1085 Hungary; 2 Department of Public Dental Health, Semmelweis University, Szentkirályi Utca 40, Budapest, 1088 Hungary

**Keywords:** Xerostomia, NBF gingival gel, Gel-type high-functional toothpaste, Radiotherapy, Chemotherapy, Sjögren’s disease

## Abstract

**Background:**

Xerostomia, or dry mouth, can be a temporary or persistent symptom resulting from various factors, such as medication use, therapeutic radiation, chemotherapy, autoimmune conditions (e.g., Sjögren’s syndrome), and hormonal imbalances. Xerostomia often leads to associated mucositis, which significantly impacts patients’ quality of life. The nano-bio-fusion (NBF) gingival gel, a gel-type functional toothpaste containing vitamins C, E, propolis, and herbal extracts in a nano-emulsion state, has shown potential in accelerating the healing of oral mucosal lesions.

**Methods:**

A total of 127 patients (102 females, 25 males) with persistent xerostomia were treated from 2018 to 2023. Of these, 32 patients were treated exclusively with NBF Gel, while 95 patients received NBF Gel in combination with other medications, such as pilocarpine. The underlying causes of xerostomia included irradiation and chemotherapy (12 patients), medication (40 patients), hormonal imbalance (28 patients), and Sjögren’s syndrome (47 patients). NBF Gel was applied 2–3 times daily to the tongue and oral mucosa. Treatment effectiveness was evaluated through physical examinations and a patient-reported scale ranging from 1 (no improvement) to 10 (complete improvement), focusing on the healing of mucosal lesions rather than saliva production.

**Results:**

Both treatment groups showed significant improvements in the healing of xerostomia-associated mucositis, particularly in severe cases with visible lesions. Patients treated with NBF Gel reported improved symptoms related to mucosal health, while those who received combination therapy also experienced reduced side effects of pilocarpine due to dose reduction. The most substantial improvements were observed in patients with drug-induced and hormonally-caused xerostomia-related mucositis. No adverse side effects from NBF Gel were reported during the study.

**Conclusion:**

NBF gingival gel proved to be beneficial in accelerating the healing of mucositis associated with xerostomia, regardless of the underlying cause, including medication use, radiotherapy, chemotherapy, hormonal imbalances, and Sjögren’s syndrome. It presents a promising adjunctive treatment to improve mucosal health and quality of life for patients suffering from xerostomia-associated mucositis.

## Background

Xerostomia, commonly known as dry mouth, can be categorized as either temporary or persistent [1, 2]. Temporary xerostomia typically occurs when salivary secretion is reduced due to short-term stimuli, such as dehydration or anxiety, and generally does not cause significant changes to the oral mucosa. In contrast, persistent xerostomia involves a more complex etiology and is often associated with a variety of underlying conditions, including medications (e.g., sedatives, antihypertensive drugs, psychiatric drugs), therapeutic radiation to the salivary glands, chemotherapy, autoimmune disorders such as Sjögren’s syndrome, and hormonal imbalances [1–3]. In Hungary, persistent xerostomia affects approximately one-third of the population, leading to a significant burden on patients’ quality of life due to associated oral complications [4].

The primary challenge in managing persistent xerostomia is that it is often difficult or impossible to eliminate the underlying cause, particularly when the salivary glands have sustained permanent damage [5–7]. Consequently, treatment is primarily symptomatic, focusing on alleviating discomfort rather than addressing the root cause. Standard interventions include the use of artificial saliva, specialized toothpastes, Metrogyl dental gel, and agents that stimulate salivary gland function, such as pilocarpine [8–11]. However, these treatments often come with side effects, and their effectiveness is limited when significant glandular damage has occurred [8, 12].

A major concern for patients suffering from xerostomia is xerostomia-associated mucositis, characterized by inflammation, ulceration, and discomfort of the oral mucosa [10, 13, 14]. The condition can significantly exacerbate the symptoms of dry mouth, leading to difficulties in speaking, eating, and swallowing, thereby further diminishing patients’ quality of life [13, 15]. The mucinous glands, which continue to produce sticky saliva even when serous gland function is compromised, do not adequately protect against mucosal injury [5–7, 16]. This inadequacy is particularly evident when major salivary glands are damaged by factors like radiation therapy or autoimmune diseases, where local treatment of the mucosa becomes critical [4–7, 14, 17].

Treatment strategies for xerostomia therefore need to be two-fold: enhancing saliva production, where possible, and providing local therapy to accelerate the healing of the damaged oral mucosa [1, 8, 13, 18–20]. While enhancing saliva production is relatively straightforward in cases without severe glandular damage (such as sialosis, sialoadenosis, or hormonal imbalance), it is less effective in situations where significant pathological damage to the salivary glands has occurred. In such cases, local treatment targeting mucosal healing becomes more essential [5–7, 17].

Nano-bio-fusion gel (NBF Gel) is a promising treatment option for xerostomia-associated mucositis [21]. NBF Gingival Gel, a gel-type functional toothpaste, contains vitamins C and E, propolis, and herbal extracts formulated in a nano-emulsion state, which enhances its infiltration into the tissue [22]. Vitamin C and Vitamin E are powerful antioxidants that promote cell growth, tissue regeneration, and maintenance of cell membranes, which are critical for healing gums and soft tissues in the oral cavity [23–25]. Propolis, a natural bee product with antibacterial, antifungal, anti-inflammatory, and immune-stimulating properties, further supports the healing process by protecting the damaged mucosa from infection and promoting regeneration [25].

Given the potential benefits of NBF Gel in accelerating the healing of xerostomia-associated mucositis, this study aimed to evaluate its effectiveness over a 5-year period in patients with persistent xerostomia caused by various underlying factors.

## Methods

The study complied with the ethical principles of the Helsinki Declaration and was approved by the Semmelweis University Regional and Institutional Committee of Science And Research Ethics (SE RKEB 182/2023). All enrolled patients signed informed consent forms.

Over 5 years (2018–2023), 127 patients (98 females, 29 males) were treated with persistent xerostomia. In 32 cases, only NBF Gel was used, while in the second part (95 patients), the gel and other medications, especially pilocarpine (0.25 to 0.5 mg t.i.d.), were applied. The distribution of cases was as follows: 12 cases due to irradiation and chemotherapy, 40 cases related to medication use, 28 cases caused by hormonal imbalance, and 47 cases associated with Sjögren’s syndrome. Patients were instructed to apply the NBF Gel twice daily, after brushing their teeth in the morning and evening, by rubbing it onto the oral mucosa, especially the tongue, with a finger, and then avoiding eating or drinking for half an hour. If other medications were also taken by the patients, they were to be used as before. The effectiveness of the treatment was assessed partly through physical examination and partly through the patients’ subjective impressions and feelings on a scale of 1 to 10 (1 = no improvement, 10 = symptoms completely resolved). Objective measurement of saliva quantity was not conducted in these cases because the NBF Gel does not increase saliva production; rather, it promotes the healing of the oral mucosa and alleviates mucositis induced by xerostomia. Additionally, any increase in saliva quantity could be attributed to the concurrent use of saliva-stimulating medications. Therefore, the effectiveness of the treatment was assessed one week after its initiation based on mucosal healing and symptom improvement. If no improvement was observed after this period, the treatment was discontinued. Patients were also questioned about any potential side effects of the NBF cream, but none were reported.

Statistical analyses were performed using IBM SPSS software, version 28 (IBM Corporation, New York, NY, USA). Data on the subjective changes in the patients’ sensations was interpreted as ordinal. Therefore, the Mann–Whitney *U* test was performed to compare the data between study groups. Differences were considered statistically significant at *p* < 0.05.

## Results

The treatment of 127 patients demonstrated that the cream alone favorably influenced the patients’ feelings and objectively the condition of the oral mucosa. In 95 cases, our patients were already receiving pilocarpine treatment, and continuing this along with NBF Gel yielded satisfactory results (Table 1). In some cases, the dose of pilocarpine tablets could be reduced, which led to a decrease in the frequency of pilocarpine-related side effects such as dizziness, blood pressure changes, nausea, and sweating. The dosage of the medication can be adjusted based on the severity of these side effects, ranging from 3 doses of 0.5 to 0.25 mg per day.

**Table 1:** The subjectivedegree of improvementdue toNBF gel application or NBF gel application and Pilocarpine medication combined

Causes of xerostomia-associated mucositis	Patients in the NBF group	Degree of improvement in the NBF group	Patients in the NBF and pilocarpine group	Degree of improvement in the NBF and pilocarpine group	Level of significance (*p*)
Irradiation + chemotherapy	-	-	12 (8 ♂ 4 ♀)	5.83 ± 0.83	-
Medication	10 (1 ♂ 9 ♀)	7.10 ± 1.45	30 (5 ♂ 25 ♀)	7.80 ± 1.94	0.140
Hormonal imbalance	6 (0 ♂ 6 ♀)	5.17 ± 2.92	22 (4 ♂ 18 ♀)	6.95 ± 0.72	0.194
Sjögren’s syndrome	16 (2 ♂ 14 ♀)	4.50 ± 1.75	31 (9 ♂ 22 ♀)	5.10 ± 1.72	0.482

Patients experiencing xerostomia-associated mucositis due to prescribed medication reported a 7.10 ± 1.45 and 7.80 ± 1.94 degree of improvement of subjective symptoms in the NBF gel and NBF Gel and Pilocarpine groups respectively. Regarding xerostomia-associated mucositis patients with hormonal imbalance, the degree of improvement was 5.17 ± 2.92 and 6.95 ± 0.72 in the NBF gel and NBF Gel and Pilocarpine groups respectively. Patients with Sjögren’s syndrome reported a 4.50 ± 1.75 and 5.10 ± 1.72 degree of improvement in the NBF gel and NBF Gel and Pilocarpine groups respectively. Neither of these differences were statistically significant.

According to the literature, the beneficial effect of NBF Gel appears within a few minutes to 1–2 days [26, 27]. In terms of patients’ subjective feelings, improvement was indeed observed immediately. However, to assess the healing (improvement) of dry, cracked mucosal lesions, it is necessary to evaluate patient-reported outcomes one week later to determine whether the improvement persists with the use of the cream. The results are summarized in Table 1. The greatest improvement could be observed in cases of drug-induced and hormonally-caused xerostomia-associated mucositis.

## Discussion

According to the results of this study, no significant differences were observed in the degree of improvement of the subjective symptoms of patients experiencing xerostomia-associated mucositis due to their prescribed medication, hormonal imbalance, and Sjögren’s syndrome. These results suggest that for these patient groups, NBF gel without pilocarpine might be sufficient to improve subjective symptoms of constant xerostomia-associated mucositis and glossodynia inter cibos. However, NBF gel did not stimulate saliva secretion. Thus, supplementing NBF Gel with pilocarpine is necessary to avoid dysphagia.

NBF Gel was launched in South Korea in early 2008 [26, 27]. NBF Gel is a highly functional gel, created for the first time by a process where nanotechnology is fused with biotechnology [26]. The result is the creation of a nano-emulsion composed of ultra (nano) particles of vitamin C, vitamin E, and propolis extract. The mouth is naturally a moist environment; thus, the contact time of topically applied medicine is limited. The nanoparticles of vitamin C, vitamin E, and propolis extract overcome this problem due to their ultra-fine size [25, 28, 29]. They are much more efficient in rapidly penetrating the cells than their regular size counterparts [21]. Once applied, the NBF gingival gel creates a film that results in increased absorption, improved clinical potency, and decreased toxicity. The gel does not contain either alcohol (no burning effect) or benzocaine (numbing effect) [26, 27].

Since 2008, we have been using the cream at the NBF Gel prescribed at the Department of Oro-Maxillofacial Surgery and Stomatology, Semmelweis University since 2008 [21]. In our previous study, we reported on its application in 68 patients. NBF Gel was used successfully in the treatment of various oral lesions, including post-operative lesions, particularly following laser surgery, diseases of the oral mucosa (aphthous ulcers, herpes, leukoplakia, lichen, glossodynia, ulcers during chemotherapy, etc.) [21]. The international literature also reports favorable results in cases of gingivitis, chronic periodontitis, desquamative gingivitis, erosive lichen planus, and diabetic lesions [23, 24, 28, 30–36].

In the medical literature, the treatment of gingival diseases often involves antibiotics and non-steroid anti-inflammatory drugs (NSAIDs) [37]. However, oral medications frequently have systemic side effects, and topical medications are usually ineffective as they are difficult to retain in the mouth [11, 16, 19]. Thus, using an effective local agent that facilitates healing is justified. The NBF Gel is rapidly absorbed at the cellular level and is competitive with all products used for local oral mucosa treatment [21, 25, 38].

Returning to the various causes of xerostomia, the consequences of salivary gland damage or just functional loss are not straightforward. During the radiation treatment of head and neck tumors, radiation damage to the maxillofacial area occurs even if the patient wears a protective mask during the treatment, albeit to a lesser extent [5, 7, 39, 40]. The result of irradiation is the destruction of glandular cells and the proliferation of fibrotic tissues in the glandular parenchyma [5]. Even if the remaining glandular tissue function is stimulated, the drying of the oral mucosa occurs, partly because the radiation directly damages the mucosa, and partly because the serous glands do not produce sufficient saliva despite stimulation [41]. Chemotherapy has similar effects as irradiation [40, 42, 43]. During treatment, ulcers may form in the mouth in addition to xerostomia, for which the NBF Gel is excellently suitable [21].

In the case of Sjögren’s syndrome (benign lymphoepithelial lesion), lymphocytes destroy epithelial cells in the ductal system of the salivary glands, resulting in dilations and small bullae in the fine primary salivary ducts where the damaged epithelial cells used to be [6, 44]. These bullae accumulate saliva, which can become retrogradely infected. Repeated inflammations further damage the glandular tissue [6, 44]. Therefore, a two-way treatment is important: enhancing saliva secretion (pilocarpine) and locally treating the dry mucosa (NBF Gel). In this way, the patients’ objective symptoms improve, and the subjective dry, sometimes burning sensation (glossodynia) greatly improves [21].

In the case of hormonal and drug-induced dry mouth, the neurohormonal balance necessary for normal saliva secretion is disrupted [10, 18, 45–47]. In the serous glands, the basal membrane surrounding the salivary-producing cells acts as a filter: sympathetic stimuli reduce its permeability (from the glandular cells to the primary salivary ducts), while parasympathetic stimuli expand the filter, allowing saliva to be discharged from the glandular cells [48].

**Fig. 1:**
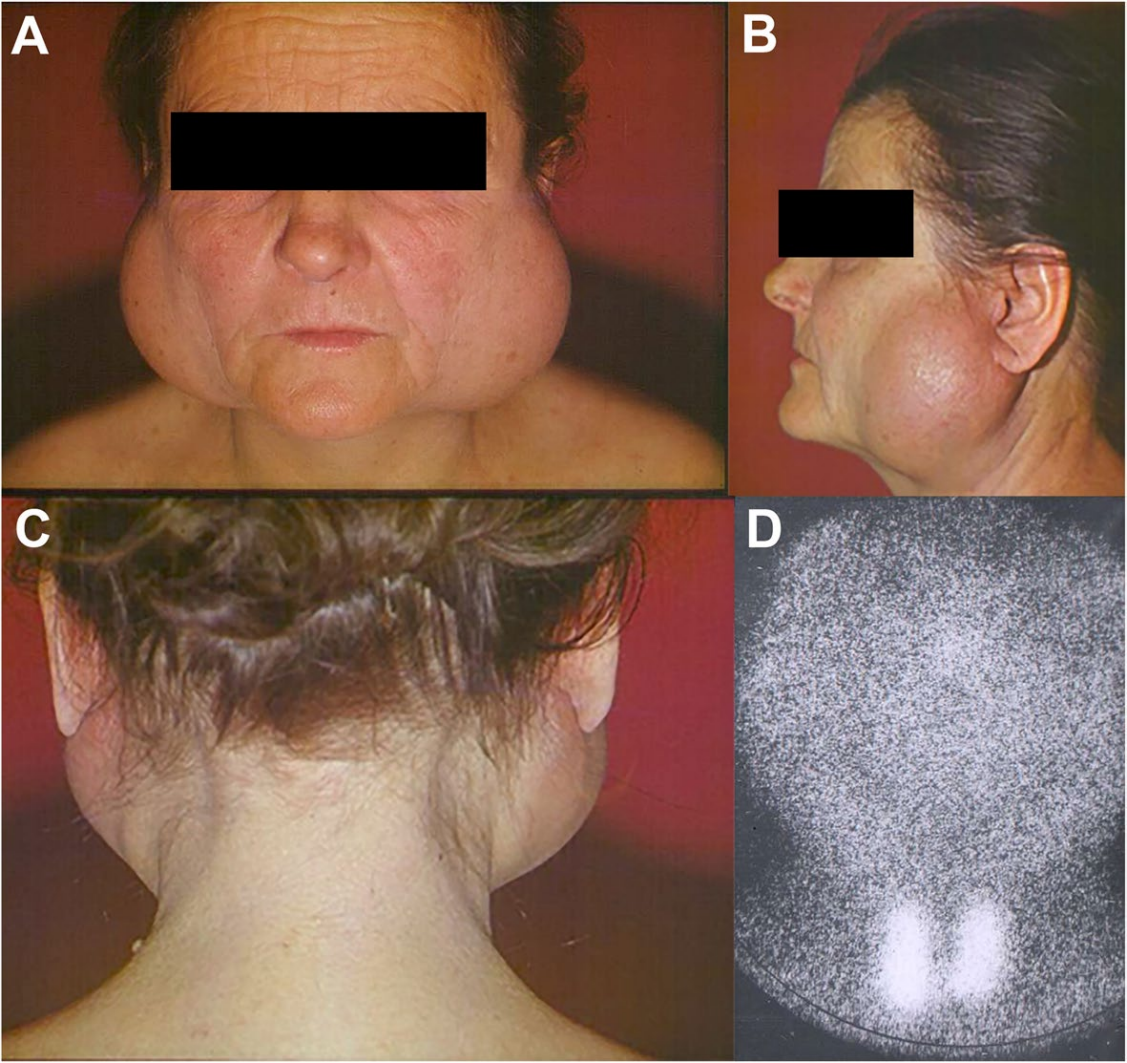
Presents the clinical features of a severe case of Sjögren’s syndrome with major lymphoid infiltration, without any remaining salivary gland tissue (**A**, **B**, and **C**). Salivary glands are not visible on the scintigraphy (**D**), only the thyroid gland

Due to its composition, the NBF cream does not improve salivation. While the cause of xerostomia-associated mucositis is the lack of saliva, the associated symptoms—such as the subjective feeling of constant dry mouth, glossodynia, oral mucosal ulcers that appear after irradiation or chemotherapy, and difficulties in swallowing during eating—are alleviated by the NBF cream [21]. Pilocarpine may be prescribed to enhance salivation, and the NBF cream complements its effect by improving subjective symptoms of xerostomia. In this study, pilocarpine and NBF cream were prescribed together. Therefore, in the present study, the effectiveness of the cream was measured by changes in the subjective feelings of the patients. There is no objective measure described in the literature for changes in the subjective comfort of xerostomia patients. In severe cases of post-irradiation xerostomia and Sjögren’s syndrome, where there is practically no functioning salivary gland tissue remaining, even with the aid of pilocarpine, it is impossible to produce more saliva. The clinical manifestation of a severe case of Sjögren’s syndrome is presented in Fig. 1. In these cases, the NBF Gel becomes even more significant as it provides the most favorable treatment for the dried-out mucosa.

Despite demonstrating promising results, this study has several limitations that should be considered when interpreting the findings. First, the study did not include a negative control group, meaning we could not directly compare the outcomes of the NBF Gel-treated group with a completely untreated group. However, we mitigated this limitation by comparing the condition of patients before and after NBF Gel treatment. In this context, the pre-treatment state served as a baseline equivalent to a “no treatment” group, allowing for some level of comparative analysis. Second, the study primarily relied on subjective measurements, such as the Visual Analogue Scale (VAS), to assess the effectiveness of NBF Gel. While subjective assessments are crucial for understanding the patient’s experience and perceived improvements, they may introduce biases or inconsistencies due to individual differences in perception and reporting. Incorporating objective measurements, such as the clinical evaluation of mucosal healing or quantitative assessments of mucosal thickness and integrity, could provide a more comprehensive understanding of the treatment’s effectiveness and reduce potential subjectivity. Third, although the study observed a reduced dosage of pilocarpine when used in combination with NBF Gel—resulting in fewer side effects—this finding was not compared to a group receiving pilocarpine alone. A direct comparison between the NBF Gel and pilocarpine combination group and a pilocarpine-only group would be necessary to better understand the additive or synergistic effects of NBF Gel on reducing pilocarpine dosage and its associated side effects. These limitations highlight the need for future studies to include negative control groups, incorporate both subjective and objective measurements, and compare combination therapies to monotherapies to strengthen the evidence for the clinical benefits of NBF Gel in treating xerostomia-associated mucositis.

## Conclusions

NBF gingival gel has shown to be effective in promoting the healing of mucositis linked to xerostomia, regardless of its cause—whether due to medication, radiotherapy, chemotherapy, hormonal changes, or Sjögren’s syndrome. The gel’s application led to a notable improvement in the symptoms of xerostomia-associated mucositis, contributing to better mucosal health and enhanced quality of life for affected patients. Importantly, the treatment with NBF Gingival Gel was well-tolerated, with no adverse side effects observed, supporting its use as a valuable adjunctive option for managing xerostomia-related mucosal conditions.

## Data Availability

The datasets used and analyzed during the current study are available from the corresponding author on reasonable request.

## Abbreviations

NBF gel: Nano-bio-fusion gel
NSAIDs: Non-steroid anti-inflammatory drugs

## References

[1] Guggenheimer J, Moore PA (2003) Xerostomia: etiology, recognition and treatment. J Am Dent Assoc. 134(1):61–69; quiz 118-119 12555958 10.14219/jada.archive.2003.0018

[2] Tanasiewicz M, Hildebrandt T, Obersztyn I (2016) Xerostomia of various etiologies: a review of the literature. Adv Clin Exp Med 25(1):199–206 26935515 10.17219/acem/29375

[3] Cassolato SF, Turnbull RS (2003) Xerostomia: clinical aspects and treatment. Gerodontology 20(2):64–77 14697016 10.1111/j.1741-2358.2003.00064.x

[4] Dézsi AJ, Erdei C, Demeter T, Kovács A, Nagy G, Mensch K, Németh O, Hermann P, Tóth G, Füst Á, Kiss EV, Kőhidai L, Zalatnai A, Márton K (2023) Prevalence of Sjögren’s syndrome in patients with dry mouth in the region of Central Hungary. Oral Dis 29(7):2756–2764 35611648 10.1111/odi.14264

[5] Bhide SA, Miah AB, Harrington KJ, Newbold KL, Nutting CM (2009) Radiation-induced xerostomia: pathophysiology, prevention and treatment. Clin Oncol (R Coll Radiol) 21(10):737–744 19833490 10.1016/j.clon.2009.09.002

[6] Jensen SB, Vissink A (2014) Salivary gland dysfunction and xerostomia in Sjögren’s syndrome. Oral Maxillofac Surg Clin North Am 26(1):35–53 24287192 10.1016/j.coms.2013.09.003

[7] Jensen SB, Vissink A, Limesand KH (2019) Reyland ME (2019) Salivary gland hypofunction and xerostomia in head and neck radiation patients. J Natl Cancer Inst Monogr. 53:lgz016 10.1093/jncimonographs/lgz016 31425600

[8] Berk L (2008) Systemic pilocarpine for treatment of xerostomia. Expert Opin Drug Metab Toxicol 4(10):1333–1340 18798702 10.1517/17425255.4.10.1333

[9] Hanchanale S, Adkinson L, Daniel S, Fleming M, Oxberry SG (2015) Systematic literature review: xerostomia in advanced cancer patients. Support Care Cancer 23(3):881–888 25322971 10.1007/s00520-014-2477-8

[10] Thakkar JP, Lane CJ (2022) Hyposalivation and xerostomia and burning mouth syndrome: medical management. Oral Maxillofac Surg Clin North Am 34(1):135–146 34598858 10.1016/j.coms.2021.08.002

[11] Hahnel S, Behr M, Handel G, Bürgers R (2009) Saliva substitutes for the treatment of radiation-induced xerostomia–a review. Support Care Cancer 17(11):1331–1343 19495809 10.1007/s00520-009-0671-x

[12] Sproll C, Naujoks C (2015) Entzündungen und obstruktive Speicheldrüsenerkrankungen Der MKG-Chirurg 3(8):128–141

[13] Radvansky LJ, Pace MB, Siddiqui A (2013) Prevention and management of radiation-induced dermatitis, mucositis, and xerostomia. Am J Health Syst Pharm 70(12):1025–1032 23719879 10.2146/ajhp120467

[14] Dose AM (1995) The symptom experience of mucositis, stomatitis, and xerostomia. In: Seminars in oncology nursing. Elsevier, Philadelphia, p 248–255 10.1016/s0749-2081(05)80005-1 8578032

[15] Choi JH, Kim MJ, Kho HS (2021) Oral health-related quality of life and associated factors in patients with xerostomia. Int J Dent Hyg 19(3):313–322 34092041 10.1111/idh.12528

[16] Davies AN (1997) The management of xerostomia: a review. Eur J Cancer Care (Engl) 6(3):209–214 9335672 10.1046/j.1365-2354.1997.00036.x

[17] Fox RI (2005) Sjögren’s syndrome. The Lancet 366(9482):321–331 10.1016/S0140-6736(05)66990-5 16039337

[18] Ito K, Izumi N, Funayama S, Nohno K, Katsura K, Kaneko N, Inoue M (2023) Characteristics of medication-induced xerostomia and effect of treatment. PLoS One 18(1):e0280224 36634078 10.1371/journal.pone.0280224 PMC9836311

[19] Malallah OS, Garcia CMA, Proctor GB, Forbes B, Royall PG (2018) Buccal drug delivery technologies for patient-centred treatment of radiation-induced xerostomia (dry mouth). Int J Pharm 541(1–2):157–166 29425763 10.1016/j.ijpharm.2018.02.004

[20] Taylor SE (2003) Efficacy and economic evaluation of pilocarpine in treating radiation-induced xerostomia. Expert Opin Pharmacother 4(9):1489–1497 12943478 10.1517/14656566.4.9.1489

[21] Szabó G, Németh Z (2010) Use of “functional tooth paste”, made with nanotechnology, in the treatment of oral mucosa diseases. Fogorv Sz 103(2):39–41 20672750

[22] Amini L, Chekini R, Nateghi MR, Haghani H, Jamialahmadi T, Sathyapalan T (2021) Sahebkar A (2021) The effect of combined vitamin C and vitamin E supplementation on oxidative stress markers in women with endometriosis: a randomized, triple-blind placebo-controlled clinical trial. Pain Res Manag 1:5529741 10.1155/2021/5529741 PMC8172324 34122682

[23] Alam MK, Ganji KK (2021) Nano-bio fusion gingival gel in the management of fixed orthodontic treatment-induced gingivitis: an empirical study. Am J Orthod Dentofacial Orthop 159(6):808–815 33773855 10.1016/j.ajodo.2020.02.022

[24] Alam MK, Ganji KK, Meshari A, Manay SM, Jamayet NB, Siddiqui AA (2021) Pain management using nano-bio fusion gel in fixed orthodontic therapy-induced gingivitis: a split-mouth design study. Appl Sci 11(23):11463

[25] Jaldin-Crespo L, Silva N, Martínez J (2022) Nanomaterials based on honey and propolis for wound healing—a mini-review. Nanomaterials 12(24):4409 36558262 10.3390/nano12244409 PMC9785851

[26] Chae C-H, Choi D-J, Shim H-Y, Byun E-S, Hong S-M, Park Y-H, Park J-W (2007) Treatment of oral soft tissue lesions and wounds with high functional tooth paste made from nanoemulsion gel. J Korean Assoc Oral Maxillofac Surg 33(6):694–700

[27] Chae C-H, Park J-W (2007) The study on the effect of nanoemulsion for the prevention and treatment of gingival inflammation. J Korean Oral Maxillofac Surg 33(6):694–700

[28] Coutinho A (2012) Honeybee propolis extract in periodontal treatment: a clinical and microbiological study of propolis in periodontal treatment. Indian J Dent Res 23(2):294 22945731 10.4103/0970-9290.100449

[29] Grange J, Davey R (1990) Antibacterial properties of propolis (bee glue). J R Soc Med 83(3):159–160 2182860 10.1177/014107689008300310 PMC1292560

[30] Chatterjee A, Sneha V (2014) Evaluate the efficacy of NBF gel as an adjunct to scaling in gingivitis—A clinical study. Periodontics 20:543–548

[31] Debnath K, Chatterjee A, Priya VS (2016) Evaluation of nano-bio fusion gel as an adjunct to scaling and root planing in chronic periodontitis: a clinico-microbiological study. J Indian Soc Periodontol. 20(5):543–548 29242691 10.4103/0972-124X.201696 PMC5676337

[32] Popovska M, Fidovski J, Mindova S, Dirjanska K, Ristoska S, Stefanovska E, Radojkova-Nikolovska V, Mitic K, Rusevska B (2016) The effects of NBF gingival gel application in the treatment of the erosive lichen planus: case report. Open Access Maced J Med Sci 4(1):158–163 27275352 10.3889/oamjms.2016.026 PMC4884239

[33] Singh N, Singh K, Kasana J (2020) The effect of nano bio fusion gel as an adjunct to conventional therapy in gingivitis patients. Int Healthcare Res J 3(10):331–334

[34] Srivastava V, Huidrom E, Meenawat A, Yadav S, Shahab Y, Kumar V (2019) Role of nano bio–fusion gel as an adjunct to scaling and root planing’. Univ J Dent Sci 5(3):09–14

[35] González-Serrano J, Serrano J, Sanz M, Torres J, Hernández G, López-Pintor RM (2023) Efficacy and safety of a bioadhesive gel containing propolis extract, nanovitamin C and nanovitamin E on desquamative gingivitis: a double-blind, randomized, clinical trial. Clin Oral Investig 27(2):879–888 35900605 10.1007/s00784-022-04653-0 PMC9889524

[36] Koo H, Cury JA, Rosalen PL, Ambrosano GM, Ikegaki M, Park YK (2002) Effect of a mouthrinse containing selected propolis on 3-day dental plaque accumulation and polysaccharide formation. Caries Res 36(6):445–448 12459618 10.1159/000066535

[37] Heasman P, Hughes F (2014) Drugs, medications and periodontal disease. Br Dent J 217(8):411–419 25342347 10.1038/sj.bdj.2014.905

[38] Suri SS, Fenniri H, Singh B (2007) Nanotechnology-based drug delivery systems. J Occup Med Toxicol 2:1–6 18053152 10.1186/1745-6673-2-16 PMC2222591

[39] Alterio D, Marvaso G, Ferrari A, Volpe S, Orecchia R, Jereczek-Fossa BA (2019) Modern radiotherapy for head and neck cancer. In: Seminars in oncology. Elsevier, Amsterdam, p 233–245 10.1053/j.seminoncol.2019.07.002 31378376

[40] Buglione M, Cavagnini R, Di Rosario F, Maddalo M, Vassalli L, Grisanti S, Salgarello S, Orlandi E, Bossi P, Majorana A, Gastaldi G, Berruti A, Trippa F, Nicolai P, Barasch A, Russi EG, Raber-Durlacher J, Murphy B, Magrini SM (2016) Oral toxicity management in head and neck cancer patients treated with chemotherapy and radiation: xerostomia and trismus (Part 2). Literature review and consensus statement. Crit Rev Oncol Hematol 102:47–54 27061883 10.1016/j.critrevonc.2016.03.012

[41] Nabil WNN, Lim RJ, Chan SY, Lai NM, Liew AC (2018) A systematic review on Chinese herbal treatment for radiotherapy-induced xerostomia in head and neck cancer patients. Complement Ther Clin Pract 30:6–13 29389481 10.1016/j.ctcp.2017.10.004

[42] Pinto VL, Fustinoni SM, Nazário ACP, Facina G, Elias S (2020) Prevalence of xerostomia in women during breast cancer chemotherapy. Rev Bras Enferm 73(suppl 4):e20190785 32965427 10.1590/0034-7167-2019-0785

[43] Zhou P, Zhou R, Yu YF, Rao MY, Wu SG (2023) Xerostomia: an easily ignored symptom induced by induction chemotherapy in patients with nasopharyngeal carcinoma. Head Neck 45(12):3024–3032 37750446 10.1002/hed.27529

[44] Manfrè V, Chatzis LG, Cafaro G, Fonzetti S, Calvacchi S, Fulvio G, Navarro Garcia IC, La Rocca G, Ferro F, Perricone C (2022) Sjögren’s syndrome: one year in review 2022. Clin Exp Rheumatol 40(12):2211–2224 36541236 10.55563/clinexprheumatol/43z8gu

[45] Barbe AG (2018) Medication-induced xerostomia and hyposalivation in the elderly: culprits, complications, and management. Drugs Aging 35(10):877–885 30187289 10.1007/s40266-018-0588-5

[46] Urien L, Jauregizar N, Lertxundi U, Fernández U, Morera-Herreras T (2024) Medication impact on oral health in schizophrenia. Med Oral Patol Oral Cir Bucal 29(1):e51–e57 37992139 10.4317/medoral.26061 PMC10765325

[47] Ślebioda Z, Szponar E (2014) Burning mouth syndrome–a common dental problem in perimenopausal women. Menopause Rev 13(3):198–202 10.5114/pm.2014.43825 PMC4520363 26327855

[48] Proctor GB (2000) (2016) The physiology of salivary secretion. Periodontol 70(1):11–25 10.1111/prd.12116 26662479

